# Sweet potato viromes in eight different geographical regions in Korea and two different cultivars

**DOI:** 10.1038/s41598-020-59518-x

**Published:** 2020-02-13

**Authors:** Yeonhwa Jo, Sang-Min Kim, Hoseong Choi, Jung Wook Yang, Bong Choon Lee, Won Kyong Cho

**Affiliations:** 1 Research Institute of Agriculture and Life Sciences, College of Agriculture and Life Sciences, Seoul National University, Seoul, 08826 Republic of Korea; 2 Crop Foundation Division, National Institute of Crop Science, Rural Development Administration, Wanju, 55365 Republic of Korea; 3 Department of Agricultural Biotechnology, College of Agriculture and Life Sciences, Seoul National University, Seoul, 08826 Republic of Korea; 4 Bioenergy Crop Research Institute, National Institute of Crop Science, Rural Development Administration, Muan, 58545 Republic of Korea

**Keywords:** Transcriptomics, Metagenomics

## Abstract

The sweet potato in the family *Convolvulaceae* is a dicotyledonous perennial plant. Here, we conducted a comprehensive sweet potato virome study using 10 different libraries from eight regions in Korea and two different sweet potato cultivars by RNA-Sequencing. Comprehensive bioinformatics analyses revealed 10 different virus species infecting sweet potato. Moreover, we identified two novel viruses infecting sweet potato referred to as Sweet potato virus E (SPVE) in the genus *Potyvirus* and Sweet potato virus F (SPVF) in the genus *Carlavirus*. Of the identified viruses, Sweet potato feathery mottle virus (SPFMV) was the dominant virus followed by Sweet potato virus C (SPVC) and SPVE in Korea. We obtained a total of 30 viral genomes for eight viruses. Our phylogenetic analyses showed many potyvirus isolates are highly correlated with geographical regions. However, two isolates of SPFMV and a single isolate of Sweet potato virus G (SPVG) were genetically distant from other known isolates. The mutation rate was the highest in SPFMV followed by SPVC and SPVG. Two different sweet potato cultivars, Beni Haruka and Hogammi, were infected by seven and five viruses, respectively. Taken together, we provide a complete list of viruses infecting sweet potato in Korea and diagnostic methods.

## Introduction

The sweet potato [*Ipomoea batatas* (L.) Lam] is a dicotyledonous perennial plant and a member of the family *Convolvulaceae*. It is currently widely grown in tropical and temperate regions in the world^1^. The sweet potato is regarded as one of the most healthful foods in the world due to its high amounts of beta-carotene, carbohydrates, and dietary fiber and diverse micronutrients^2,3^. It is a very fast-growing vine plant and is usually vegetatively propagated by cutting a piece of a runner of sweet potato approximately 30 cm in length^4^. According to the FAO, in 2017, China was the largest sweet potato producer (72,031,782 tons), followed by several African countries, such as Malawi (5,472,013 tons), the United Republic of Tanzania (4,244,370 tons), and Nigeria (4,013,786 tons). In Korea, the sweet potato has been cultivated since the end of the 18th century, and it is the third most important crop plant in South Korea based on production (331,514 tons)^5^. In general, the tuberous roots and stems of the sweet potato are used as different food materials in Korea.

Due to their vegetative propagation, most sweet potato cultivars are severely infected by various pathogens. Of the known pathogens, more than 30 different viruses infecting sweet potato have been identified so far^6^. The most frequently identified viruses are DNA viruses such as *Sweet potato leaf curl virus* (SPLCV) in the genus *Begomovirus* in the family *Geminiviridae*, followed by RNA viruses in the family *Potyviridae*^7^. Examples of well-known RNA viruses infecting sweet potato include *Sweet potato feathery mottle virus* (SPFMV), *Sweet potato virus C* (SPVC), *Sweet potato virus G* (SPVG), *Sweet potato virus 2* (SPV2), *Sweet potato latent virus* (SPLV), and *Sweet potato mild mottle virus* (SPMMV) in the family *Potyviridae*; *Sweet potato chlorotic fleck virus* (SPCFV) in the family *Flexiviridae*; and *Sweet potato chlorotic stunt virus* (SPCSV) in the genus *Crinivirus* in the family *Closteroviridae*^6^. SPLCV is transmitted by vegetative propagation and the insect vector whitefly (*Bemisia tabaci*)^8^, whereas potyviruses infecting sweet potato are also transmitted by various insect vectors including *Myzus persicae* (Sulzer)^9^. Many previous studies have demonstrated that multiple viruses such as begomoviruses and potyviruses frequently coinfect most sweet potato cultivars grown in fields, resulting in the reduction of sweet potato yield^10–12^. Moreover, the coinfection of potyviruses in sweet potato has been found to be common^10^. Vegetative propagation and insect vectors might be two main reasons for the coinfection of viruses in sweet potato.

In Korea, several studies have examined viruses infecting sweet potato^13–16^. According to the previous studies, many sweet potato germplasm collections and sweet potato cultivars grown in fields in Korea are often coinfected by different viruses^14^. At least eight different virus species infecting sweet potato have been reported based on RT-PCR assays^16^. The most frequently identified viruses infecting sweet potato in Korea are two potyviruses, SPFMV and SPVC^14,16^.

With the rapid development of next-generation sequencing (NGS) technologies, NGS-based approaches enable us to facilitate the identification of viruses, virus diagnostics, and the assembly of virus genomes^17–20^. For instance, the NGS technique is widely applied in the study of viruses infecting sweet potato by RNA-Sequencing (RNA-Seq)^17,21^ and small RNA-Seq^22,23^. Using the Illumina MiSeq platform, two DNA viruses including *Sweet potato mosaic virus* (SPMaV) and *Sweet potato leaf curl Sao Paulo virus* (SPLCSPV) in the genus *Begomovirus* and two RNA viruses including SPFMV and SPCSV were identified in South Africa^17^. In China, 11 virus species including SPVG and SPFMV have been identified from 219 samples in 10 regions in southern China by small RNA-Seq^23^.

Although studies associated with viruses infecting sweet potato have been intensively conducted, there has not yet been a comprehensive study using NGS techniques for sweet potato viromes. As shown previously in our studies, RNA-Seq is a very powerful tool to study viromes in diverse plants^24–27^. Here, we conducted a study of sweet potato viromes by RNA-Seq. For that, 10 different libraries were prepared from leaf samples collected from eight regions in Korea and two different sweet potato cultivars. Comprehensive bioinformatics analyses revealed the complexity of sweet potato viromes in different geographical regions and different cultivars. Moreover, we identified two novel viruses infecting sweet potato and two virus variants that might be ancestors of SPFMV and SPVG.

## Results

### Collection of leaf samples for sweet potato virome study

**Figure 1:**
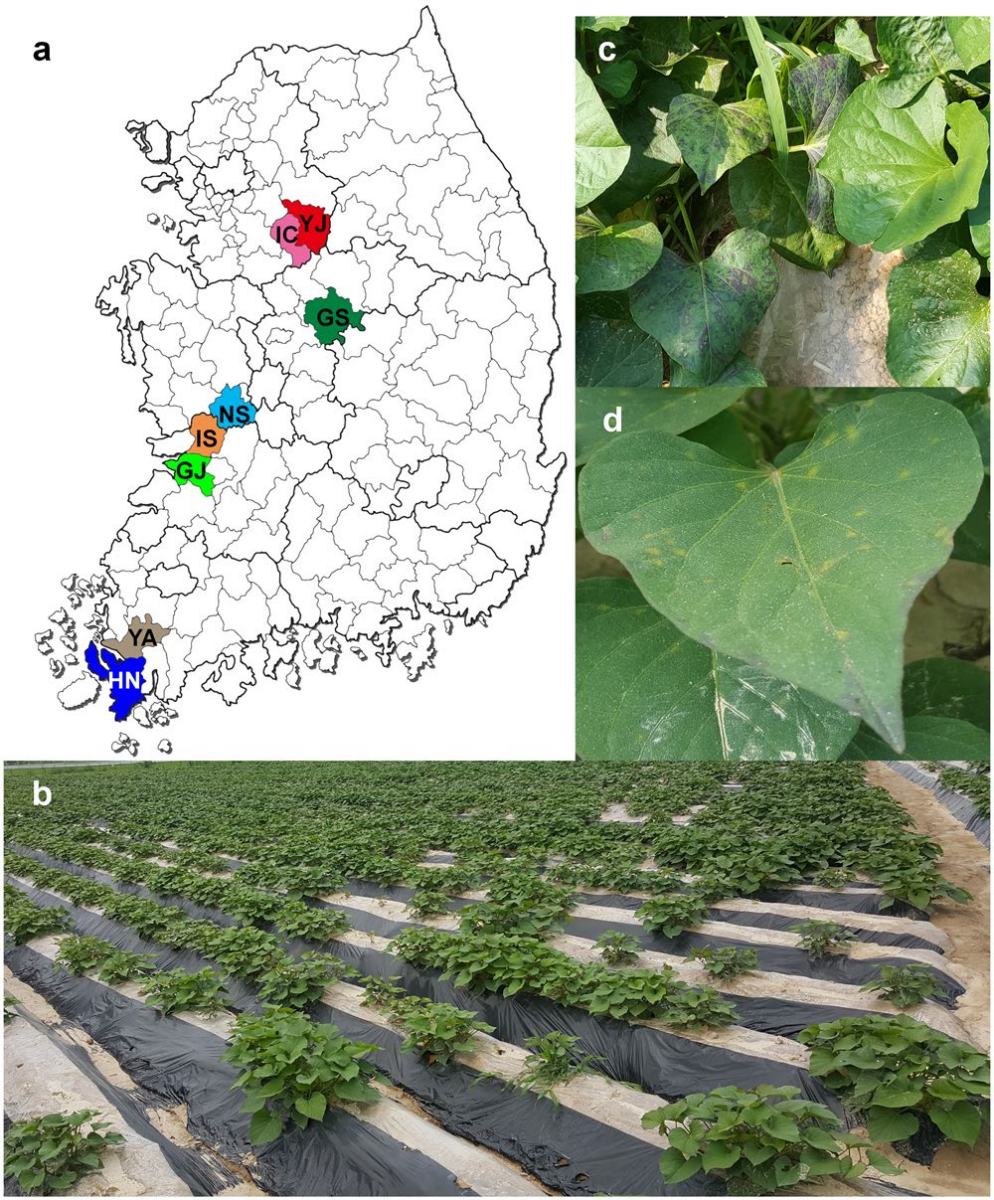
Geographical regions of collected sweet potato samples in Korea and viral disease symptoms in sweet potato plants. (**a**) A map displaying eight different geographical regions in Korea in which sweet potato samples were collected. Each region is indicated by a different color with the abbreviated region name. Full names of geographical regions can be found in Table 1. (**b**) Sweet potato plants grown in field covered by poly mulch sheets to protect soil. (**c**) Sweet potato plants displaying leaf malformation and purpling. (**d**) Leaf spots on sweet potato leaf.

In order to identify viruses infecting sweet potato in Korea, we collected leaf samples from eight different geographical regions in 2016 and 2017 (Table 1 and Fig. 1a). All sweet potato plants were grown in fields, as shown in Fig. 1b. We collected leaf samples showing disease symptoms (Fig. 1c,d). Samples were pooled based on the collected regions. In addition, we collected two representative cultivars referred to as “Beni Haruka” and “Hogammi,” which were widely cultivated in Yeoju, Korea. A total of 357 samples were subjected to RNA-Seq. Ten different libraries for RNA-Seq were prepared and paired-end sequenced by the HiSeq2000 system. For simplicity, we named the libraries based on geographical regions and cultivar names (Table 2). For instance, a library from “Hogammi” cultivated in Yeoju was referred to as YJ-H.

**Table 1:** Detailed information of sweet potato samples for sweet potato viromes in Korea.

Index	Geographical region	Cultivar	No. of samples	Name of library	Sampling year
1	Gimje	Mixed	5	GJ	2016
2	Goesan	Mixed	57	GS	2017
3	Haenam	Mixed	5	HN	2016
4	Icheon	Mixed	68	IC	2017
5	Iksan	Mixed	5	IS	2016
6	Nonsan	Mixed	5	NS	2016
7	Yeongam	Mixed	5	YA	2016
8	Yeoju	Mixed	5	YJ	2016
9	Yeoju	Benny Haruka	147	YJ-B	2017
10	Yeoju	Hogammi	55	YJ-H	2017

Geographical regions were abbreviated and used for library names. Sweet potato leaf samples were pooled according to geographical regions. The sweet potato cultivar names of mixed samples were unknown. Two known sweet potato cultivars, “Beni Haruka” and “Hogammi,” grown in Yeoju were used. Samples were collected in May 2016 and May 2017.

**Table 2:** Summary of raw sequence data for RNA-Seq and SRA accession number.

Index	Name of library	Total read bases (bp)	No. of total reads	GC(%)	SRA accession number
1	GJ	2,516,015,242	24,911,042	44	SRR8489804
2	GS	5,286,705,418	52,343,618	45.18%	SRR8489848
3	HN	1,690,693,742	16,739,542	43.698	SRR8492257
4	IC	5,246,048,272	51,941,072	45.72%	SRR8492258
5	IS	2,648,636,524	26,224,124	46.68	SRR8492261
6	NS	2,668,487,872	26,420,672	46.823	SRR8492260
7	YA	2,859,219,504	28,309,104	45.877	SRR8492261
8	YJ	2,671,667,958	26,452,158	46.57	SRR8492262
9	YJ-B	5,600,617,862	55,451,662	45.08%	SRR8492263
10	YJ-H	5,257,315,832	52,052,632	46.57%	SRR8492264

Library names were abbreviated according to sampling regions. Two fastq files containing paired-end sequence reads were deposited in the SRA with the corresponding accession number. PRJNA517178 is the SRA accession number for this project.

### Transcriptome assembly and virus identification

**Figure 2:**
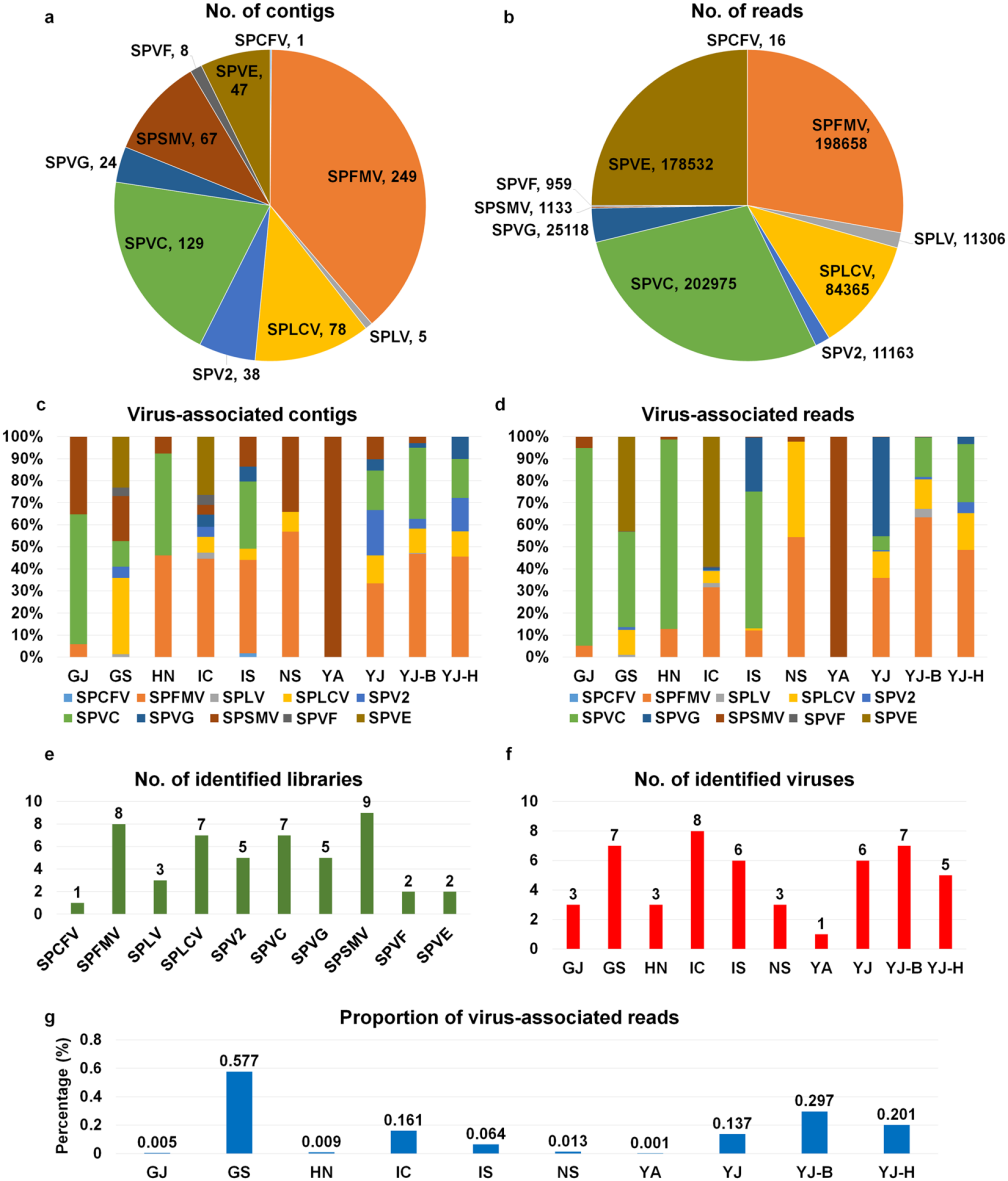
Identification of viruses infecting sweet potato and proportion of identified viruses in each library. Pie chart illustrating proportion of identified viruses from all 10 libraries based on virus-associated contigs (**a**) and virus-associated reads (**b**). Bar graphs illustrating proportion of identified viruses in each library based on virus-associated contigs (**c**) and virus-associated reads (**d**). (**e**) Number of identified libraries for individual virus. (**f**) Number of identified viruses in each library. (**g**) Proportion of virus-associated reads as compared to total sequence reads in each library.

Raw sequence reads from 10 libraries were individually *de novo* assembled using the Trinity program. The number of assembled contigs (transcripts) ranged from 65,866 (HN) to 187,838 (NS) (Table 3). The contigs obtained from each library were used for a BLASTN search against the plant virus reference genome sequences derived from the viral genome database (https://www.ncbi.nlm.nih.gov/genome/viruses/). Based on the BLASTN search using assembled contigs, we obtained a total of 646 virus-associated contigs representing 10 different sweet potato viruses from 10 sweet potato transcriptomes (Table 4). Based on the number of virus-associated contigs, SPFMV (249 contigs) was the dominant virus infecting sweet potato followed by SPVC (129 contigs), SPLCV (78 contigs) (Fig. 2a), while based on virus-associated reads, SPVC (202,975 reads) was the major virus infecting sweet potato followed by SPFMV (198,658 reads) and SPVE (Sweet potato virus E) (178,532 reads) (Fig. 2b). We examined the proportion of identified viruses in each library based on virus-associated contigs (Fig. 2c) and reads (Fig. 2d). According to virus-associated contigs, SPFMV was the major virus in seven libraries including HN, IC, IS, NS, YJ, YJ-B, and YJ-H (Fig. 2c). Based on virus-associated reads, SPVC was the major virus in four libraries including GJ, GS, HN, and IS, while SPFMV was the dominant virus in NS, YJ-B, and YJ-H (Fig. 2d). SPVE and SPVG were the dominant viruses in IC and YJ, respectively. From the YA library, only SPSMV was identified.

**Table 3:** Summary of *de novo* transcriptome assembly by Trinity.

Index	Name of library	Total trinity transcripts	Percent GC	Contig N50	Median contig length	Average contig	Total assembled bases
1	GJ	73371	43.87	970	433	672.38	49333287
2	GS	132418	41.82	1507	535	902.08	119451072
3	HN	65866	44.43	838	405	612.85	40365766
4	IC	113660	42.62	1338	479	810.88	92164062
5	IS	176281	43.44	1047	434	694.65	122454002
6	NS	187838	44.19	982	420	665.55	125015489
7	YA	122883	42.01	1542	531	910.16	111843271
8	YJ	103809	42.96	1304	486	809.75	84058928
9	YJ-B	128922	42.16	1369	479	823.45	106161259
10	YJ-H	112891	42.46	1361	522	846.01	95507393

Raw sequence reads in each library were *de novo* assembled by Trinity. Statistical summaries of assembled contigs were obtained by Trinity toolkit using the “TrinityStats.pl” command.

**Table 4:** Information of virus-associated contigs for viruses identified in sweet potato in Korea.

Index	Virus name	Accession No.	Size of genome	Genome type	Abbreviation	GJ	GS	HN	IC	IS	NS	YA	YJ	YJ-B	YJ-H
1	*Sweetpotato chlorotic fleck virus*	NC_006550.1	9,104	RNA	SPCFV					1					
2	*Sweetpotato feathery mottle virus*	NC_001841.1	10,820	RNA	SPFMV	1		6	49	25	25		13	94	36
3	*Sweetpotato latent virus*	NC_020896.1	10,081	RNA	SPLV		1		3					1	
4	*Sweetpotato leaf curl virus DNA A*	NC_004650.1	2,828	DNA	SPLCV		27		8	3	4		5	22	9
5	*Sweetpotato virus 2*	NC_017970.1	10,731	RNA	SPV2		4		5				8	9	12
6	*Sweetpotato virus C*	NC_014742.1	10,820	RNA	SPVC	10	9	6		18			7	65	14
7	*Sweetpotato virus G*	NC_018093.1	10,798	RNA	SPVG				6	4			2	4	8
8	*Sweetpotato symptomless mastrevirus 1*	NC_034630.1	2,886	DNA	SPSMV	6	16	1	5	8	15	6	4	6	
9	Sweetpotato virus F	MH388501	9,122	RNA	SPVF		3		5						
10	Sweetpotato virus E	MH388502	10,890	RNA	SPVE		18		29						

Assembled contigs were subjected to MEGABLAST against the plant viral database. Numbers indicate corresponding virus-associated contigs in each library. Virus name, accession number of reference viral genome, and abbreviated virus name for identified virus can be found. RNA and DNA indicate genome type of individual virus. SPVE and SPVF were novel viruses identified in this study.

We examined the number of libraries for the individual identified virus (Fig. 2e). SPSMV was the most frequently identified virus (nine libraries), whereas SPCFV was identified from a single library. SPFMV (eight libraries), SPLCV (seven libraries), and SPVC (seven libraries) were also frequently identified. The number of identified viruses in each library ranged from one (YA) to eight (IC) (Fig. 2f). In the case of libraries from the YJ region, five (YJ-H) to seven (YJ-B) viruses were identified.

Next, we examined the proportion of virus-associated reads in each library (Fig. 2g). The proportion of virus-associated reads ranged from 0.001% (YA) to 0.577% (GS). Libraries from the GS, IC, and YJ regions contained many virus-associated reads compared to other regions.

### Identification of two novel viruses infecting sweet potato

**Figure 3:**
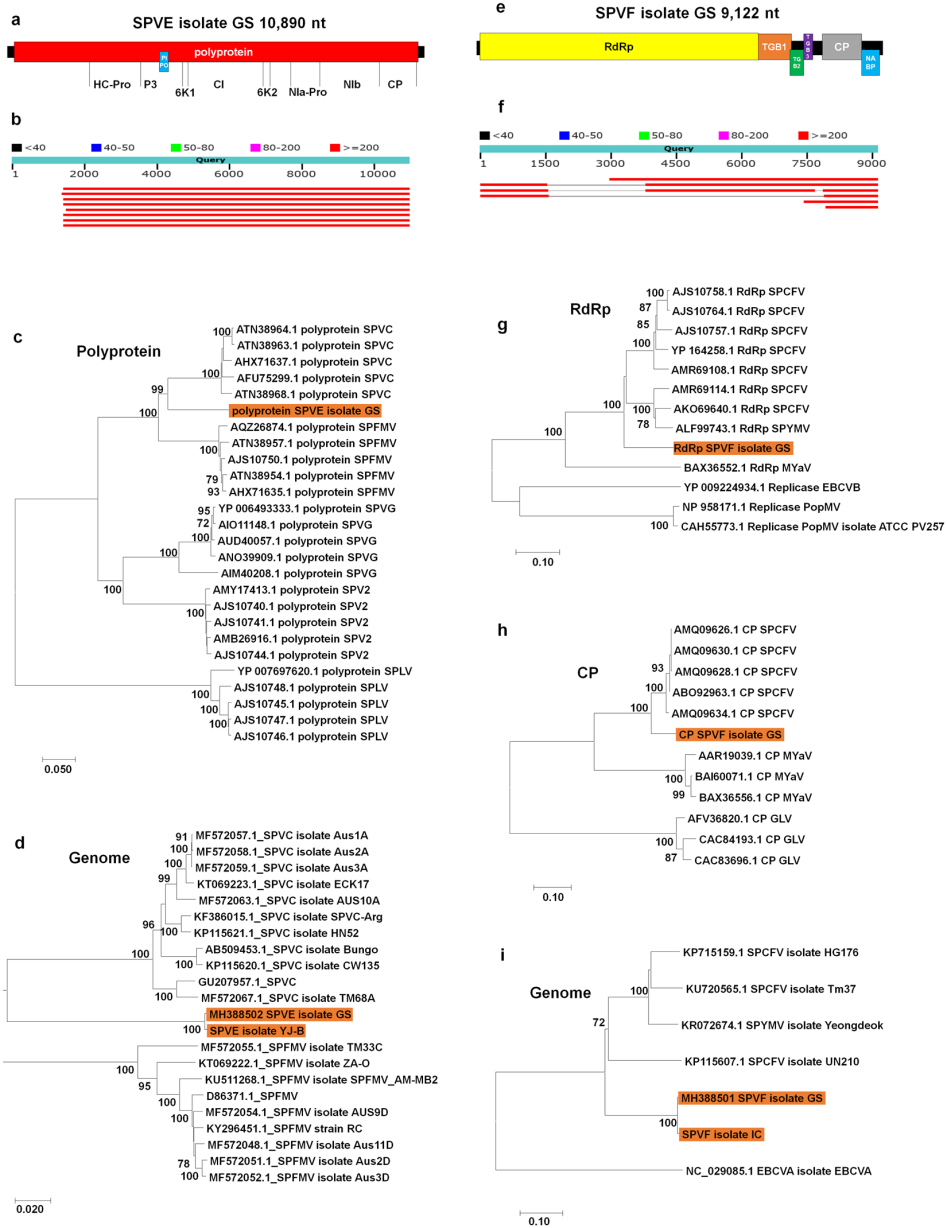
Genome organization and phylogenetic analyses of two novel viruses, SPVE and SPVF. (**a**) Genome organization of SPVE isolate GS. Polyprotein cleavage sites were also indicated with respective protein names. (**b**) BLASTN results using complete genome of SPVE against NCBI’s NT database. (**c**) Maximum likelihood phylogenetic tree of polyprotein amino acid sequences for SPVC, SPVE, SPFMV, SPVG, SPV2, and SPLV. (**d**) Maximum likelihood phylogenetic tree of genome sequences for SPVC, SPFMV, SPVE, and SPFMV. Two genome sequences for SPVE isolates GS and YJ-B were included in the phylogenetic construction. (**e**) Genome organization of SPVF isolate GS. (**f**) BLASTN results using complete genome of SPVF against NCBI’s NT database. (**g**) Maximum likelihood phylogenetic tree of RdRp amino acid sequences for SPCFV, SPYMV, SPVF, Melon yellowing-associated virus (MYaV), Elderberry carlavirus B (EBCVB), and Poplar mosaic virus (PopMV). (**h**) Maximum likelihood phylogenetic tree of CP amino acid sequences for SPCFV, SPVF, MYaV, and Garlic latent virus (GLV). (**i**) Maximum likelihood phylogenetic tree of genome sequences for SPCFV, SPYMV, and SPVF. EBCVA was used as an outgroup. Two genome sequences for SPVF isolates GS and IC were included in the phylogenetic construction. For the phylogenetic tree construction, all available protein or genome sequences homologous to SPVE or SPVF were retrieved from GenBank based on BLASTP and BLASTN searches, respectively. Accession number, isolate name, and virus name were described. Orange color indicates SPVE or SPVF. We used bootstrap replication values of 1,000, and bootstrap values over 70% are shown.

SPVE isolate GS with a single-stranded RNA virus of 10,890 nt was assembled from the GS library. SPVE isolate GS contained two ORFs encoding a polyprotein and pretty interesting potyviridae ORF (PIPO) (Fig. 3a). A BLASTN search against the NT database revealed that SPVE isolate GS shared 86% coverage and 77% nucleotide identity with SPVC isolate ECK17 (KT069223.1) identified from South Africa (Fig. 3b). The phylogenetic tree based on polyprotein amino acid sequences showed that SPVE isolate GS belongs to the same clade as five SPVC isolates, which are members of the genus *Potyvirus* in the family *Potyviridae* (Fig. 3c). We identified SPVE from GS and YJ-B; however, the genome of SPVE isolate YJ-B was incomplete. The phylogenetic tree based on complete genome sequences for SPVE, SPVC, and SPFMV showed that SPVE is distantly related with SPVC and SPFMV. Species demarcation criteria for the genus *Potyvirus* for the complete ORF are <76% nucleotide identity and <82% amino acid identity. Complete ORF of SPVE showed 73.88% nucleotide identity and 79.60% amino acid identity, demonstrating that SPVE is a new species in the genus *Potyvirus* (Table S1 and Fig. 3d).

We identified two novel viruses tentatively named SPVE and Sweet potato virus F (SPVF) from the sweet potato transcriptomes (Fig. 3). The SPVF isolate GS with a single-stranded RNA genome of 9,122 nucleotides (nt) was assembled from the GS library. SPVF isolate GS contained six open reading frames (ORFs) encoding RNA-dependent RNA polymerase (RdRp), three triple gene block (TGB) proteins, one coat protein (CP), and one nucleic acid-binding protein (NABP) (Fig. 3e). A BLASTN search against the nucleotide (NT) database of the National Center for Biotechnology Information (NCBI) revealed that SPVF isolate GS shared 67% coverage and 76% nucleotide identity with the SPCFV isolate UN210 (KP115607.1) identified from Korea (Fig. 3f). The phylogenetic trees based on RdRp (Fig. 3g) and CP (Fig. 3h) amino acid sequences showed that SPVF belongs to the same clade as SPCFV, which is a member of the genus *Carlavirus* in the family *Betaflexiviridae*. We identified SPVF from the GS and IC libraries; however, the genome of SPVF isolate IC was incomplete. The phylogenetic tree using genome sequences for SPVF isolates GS and IC and three SPCFV isolates showed that two SPVF were grouped together. Species demarcation criteria for the genus *Carlavirus* are 72% nt identity or 80% aa identity between their respective CP or polymerase genes. RNA-dependent RNA polymerase (RdRp) and coat protein (CP) for SPVF showed 78.41% and 81.89% amino acid identity, respectively, demonstrating that SPVF is a new species in the genus *Carlavirus* (Table S1 and Fig. 3i).

### Complete genomes of viruses infecting sweet potato and phylogenetic analyses

From sweet potato transcriptomes, we assembled genomes of several viruses infecting sweet potato. We assembled 12 nearly complete genomes covering ORFs for seven viruses including SPFMV, SPLCV, SPLV, SPVC, SPVE, SPVF, and SPVG (Table 5). Except for SPLCV and SPVF, all viruses belonged to the genus *Potyvirus*. In addition, we obtained several partial sequences for the identified sweet potato viruses.

**Table 5:** Detailed information for 12 assembled virus genomes from sweet potato transcriptomes.

Index	Virus name	Abbreviation	Family	Genus	Isolate name	Genome size	Accession No.
1	*Sweetpotato feathery mottle virus*	SPFMV	*Potyviridae*	*Potyvirus*	IC	10812	MH388493
2	*Sweetpotato feathery mottle virus*	SPFMV	*Potyviridae*	*Potyvirus*	YJ	10877	MH388494
3	*Sweetpotato feathery mottle virus*	SPFMV	*Potyviridae*	*Potyvirus*	YJ-B	10714	MH388495
4	*Sweetpotato leaf curl virus DNA A*	SPLCV	*Geminiviridae*	*Begomovirus*	GS	2829	MH388496
5	*Sweetpotato latent virus*	SPLV	*Potyviridae*	*Potyvirus*	YJ-B	10065	MH388497
6	*Sweetpotato virus C*	SPVC	*Potyviridae*	*Potyvirus*	GS	10827	MH388498
7	*Sweetpotato virus C*	SPVC	*Potyviridae*	*Potyvirus*	IC	10798	MH388499
8	*Sweetpotato virus C*	SPVC	*Potyviridae*	*Potyvirus*	IS	10796	MH388500
9	Sweetpotato virus F	SPVF	*Betaflexiviridae*	*Carlavirus*	GS	9122	MH388501
10	Sweetpotato virus E	SPVE	*Potyviridae*	*Potyvirus*	GS	10890	MH388502
11	*Sweetpotato virus G*	SPVG	*Potyviridae*	*Potyvirus*	IS	10785	MH388503
12	*Sweetpotato virus G*	SPVG	*Potyviridae*	*Potyvirus*	YJ	10770	MH388504

We assembled 12 complete genomes for seven virus species infecting sweet potato by RNA-Seq. The taxonomy information of the individual virus, such as family and genus, was provided. The isolate name for each virus was derived from the library and cultivar names. Genome sequences of all 12 viruses can be retrieved from GenBank with respective accession numbers.

**Figure 4:**
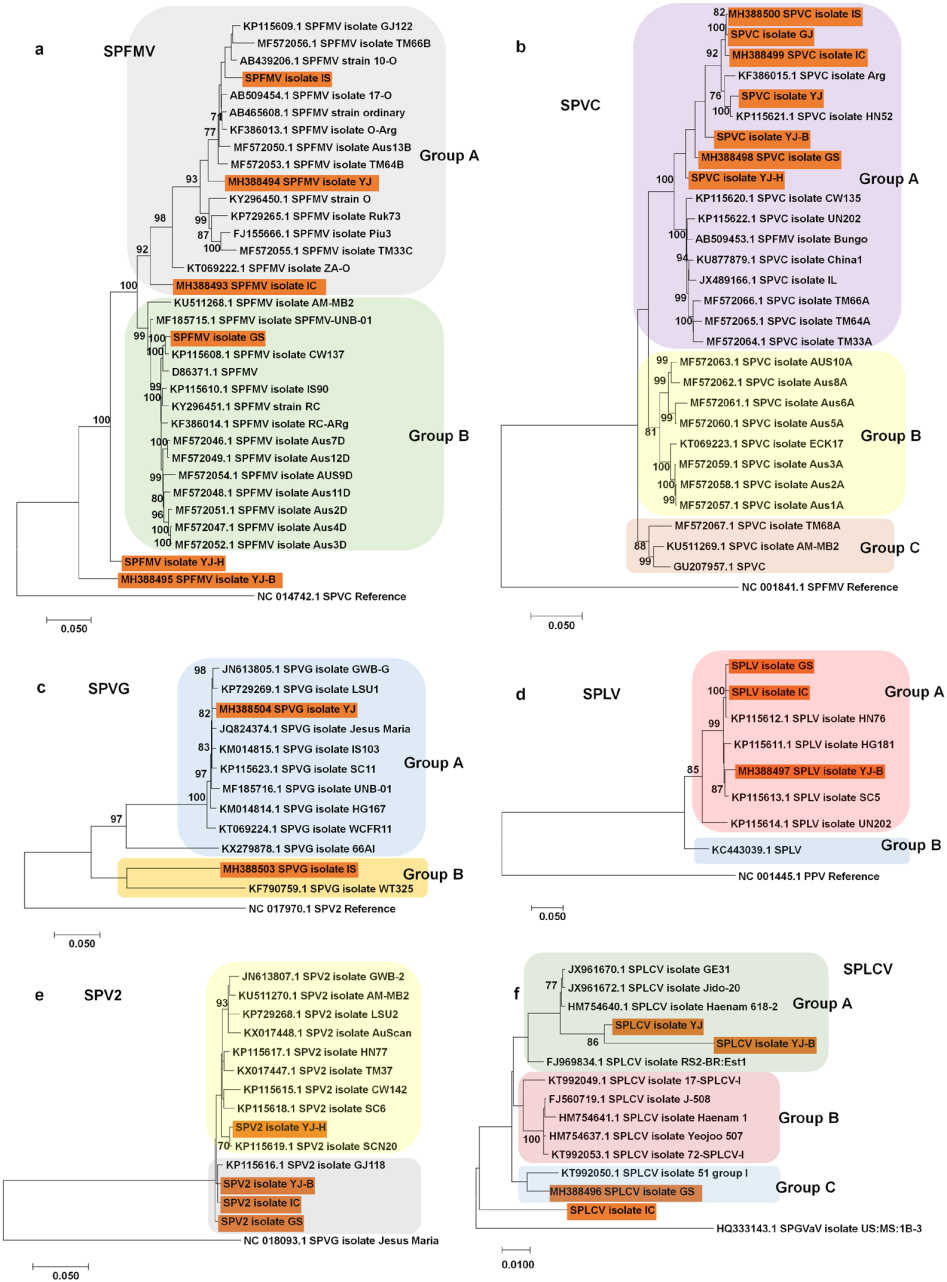
Phylogenetic analyses of SPFMV, SPVC, SPVG, SPLV, SPV2, and SPLCV isolates. (**a**) Maximum likelihood phylogenetic tree of genome sequences for 33 SPFMV isolates including six isolates from this study indicated by orange color. SPVC was used as an outgroup. (**b**) Maximum likelihood phylogenetic tree of genome sequences for 28 SPVC isolates including seven isolates in this study indicated by orange color. SPFMV was used as an outgroup. (**c**) Maximum likelihood phylogenetic tree of genome sequences for 12 SPVG isolates including two isolates in this study indicated by orange color. SPV2 was used as an outgroup. (**d**) Maximum likelihood phylogenetic tree of genome sequences for eight SPLV isolates including three isolates in this study indicated by orange color. *Plum pox virus* (PPV) was used as an outgroup. (**e**) Maximum likelihood phylogenetic tree of genome sequences for 14 SPV2 isolates including four isolates in this study indicated by orange color. SPVG was used as an outgroup. (**f**) Maximum likelihood phylogenetic tree of genome sequences for 14 SPLCV isolates including four isolates in this study indicated by orange color. SPGVaV was used as an outgroup. For phylogenetic analyses, we retrieved only complete genome sequences for each virus from GenBank based on a BLASTN search. We used not only complete viral genome sequences with respective accession numbers but also nearly complete viral genome sequences (Table S2) without accession numbers in this study. Accession number, isolate name, and virus name were described. Orange color indicates virus genomes obtained from this study. We used bootstrap replication values of 1,000, and bootstrap values over 70% are shown.

In order to reveal the phylogenetic relationships of the identified viruses, we generated phylogenetic trees based on genome sequences (Table S2 and Fig. 4). The phylogenetic tree of SPFMV showed two well-defined groups, Group A and Group B (Fig. 4a). Group A contained three SPFMV isolates, IS, YJ, and IC, while Group B included a single SPFMV isolate GS. Interestingly, two SPFMV isolates, YJ-H and YJ-B, were genetically distant from other known SPFMV isolates. According to the phylogenetic tree, SPFMV isolate YJ-B is a common ancestor of known SPFMV isolates. Genome sequences of seven SPVC isolates in this study were used for phylogenetic tree construction (Fig. 4b). The phylogenetic tree of SPVC showed three defined groups. All SPVC isolates in this study belonged to Group A containing isolates from Korea, China, and Australia. The phylogenetic tree of SPVG revealed two groups of SPVG isolates (Fig. 4c). According to the phylogenetic tree, two SPVG isolates, IS and WT325, in Group B were distantly related to those in Group A. The phylogenetic tree using genome sequences of SPLV isolates revealed that three SPLV isolates were very closely related (Fig. 4d). In the case of SPV2, all four SPV2 isolates in this study were closely related (Fig. 4e). The phylogenetic tree of SPLCV displayed three defined groups (Fig. 4f). Group A included two SPLCV isolates, YJ and YJ-B, whereas Group C contained only SPLCV isolate GS. In particular, SPLCV isolate IC was genetically distant from other SPLCV isolates.

### Mapping of raw sequence reads and mutation rates of identified viruses

**Figure 5:**
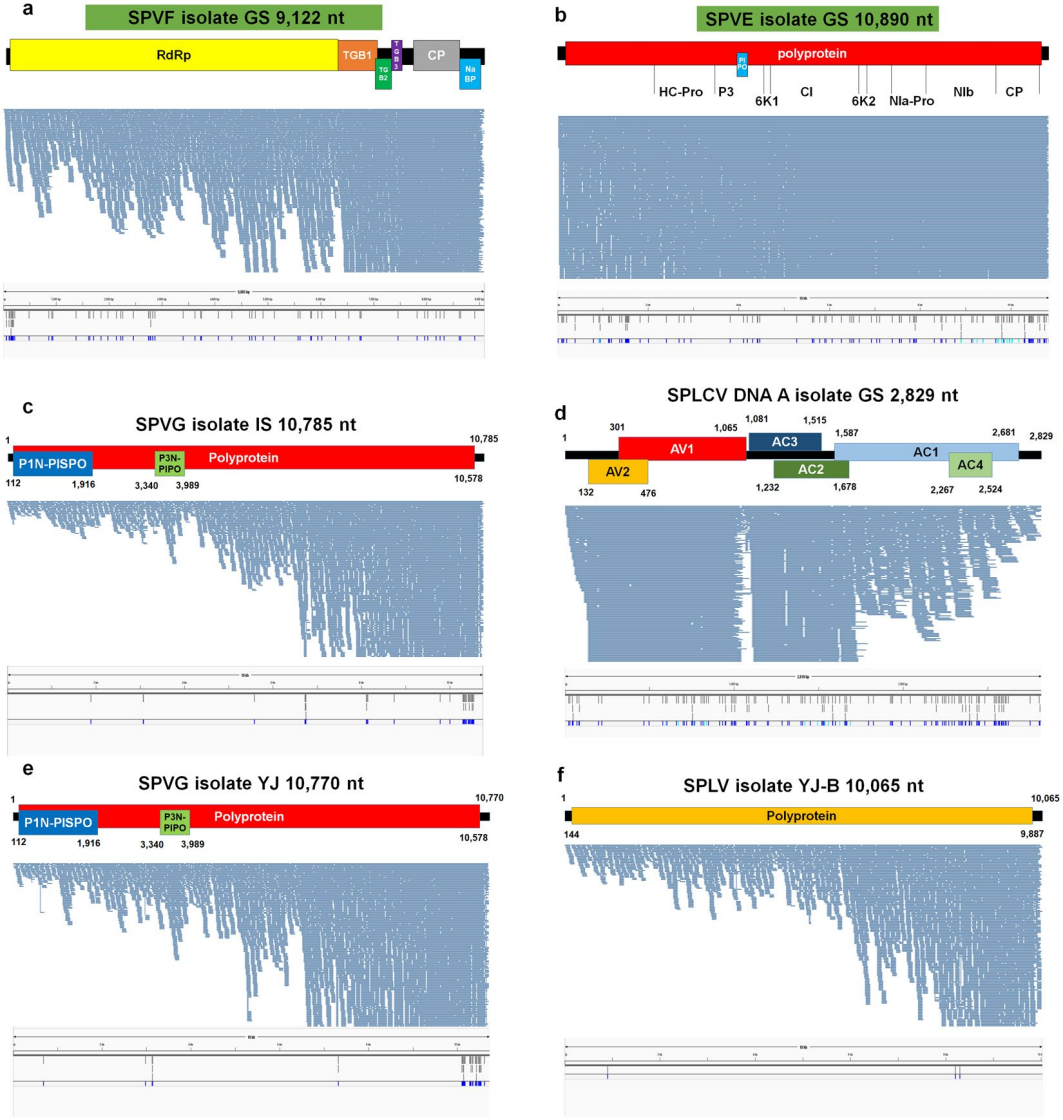
Viral genome assembly and SNP analyses for six assembled viruses including SPVF, SPVE, SPVG, SPLCV, and SPLV. Genome organization of assembled virus genome, mapping results of sequence reads on the assembled virus genome, and positions of identified SNPs for SPVF isolate GS (**a**), SPVE isolate GS (**b**) SPVG isolate IS (**c**), SPLCV DNA A isolate GS (**d**), SPVG isolate YJ (**e**), and SPLV isolate YJ-B (**f**) were visualized. Based on assembled virus-associated contigs and mapping of sequence reads on the reference virus genome, viral genomes were obtained. Positions of individual ORFs were indicated. In addition, mapping results on the assembled individual virus genome were used for SNP identification. The positions of identified SNPs in each virus genome were visualized by the Tablet program.

**Figure 6:**
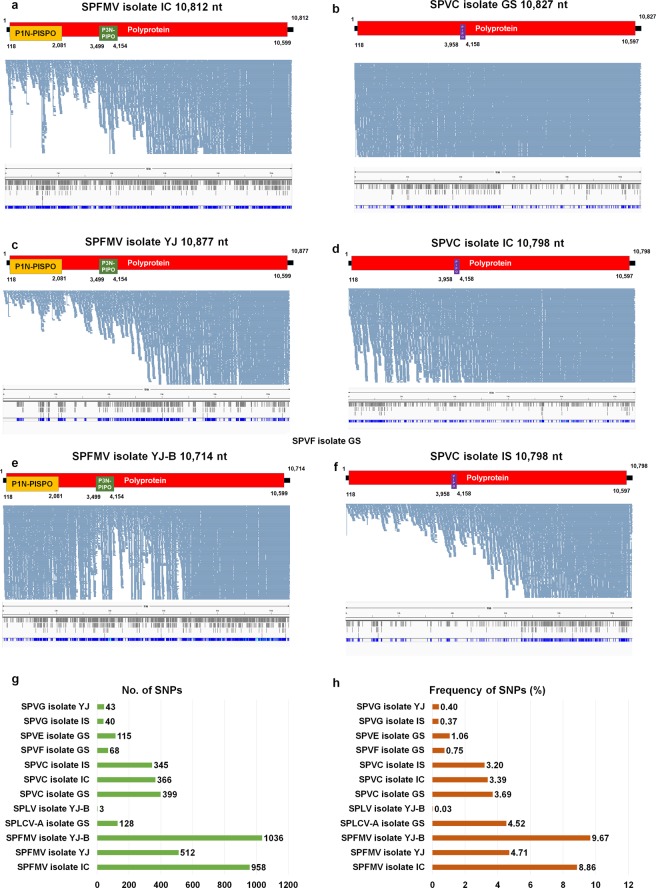
Viral genome assembly and SNP analyses for three SPFMV isolates and three SPVC isolates and mutation analyses of 12 virus genomes. Genome organization of assembled virus genome, mapping results of sequence reads on the assembled virus genome, and positions of identified SNPs for SPFMV isolate IC (**a**), SPVC isolate GS (**b**) SPFMV isolate YJ (**c**), SPVC isolate IC (**d**), SPFMV isolate YJ-B (**e**), and SPVC isolate IS (**f**) were visualized. Number of identified SNPs **(g)** and frequency of SNPs for the 12 virus genomes. Based on assembled virus-associated contigs and mapping of sequence reads on the reference virus genome, viral genomes were obtained. Positions of individual ORFs were indicated. In addition, mapping results on the assembled individual virus genome were used for SNP identification. The positions of identified SNPs in each virus genome were visualized by the Tablet program.

Viruses evolve faster than other organisms and exhibit strong genetic variation within the infected host. To determine virus mutations, we mapped raw sequence reads on the 12 virus genomes in which nearly complete genomes were assembled from this study (Figs. 5 and 6). Using SAMtools, we conducted variant calling for individual virus genomes resulting in the identification of single nucleotide polymorphisms (SNPs).

Firstly, we examined the mapping patterns of sequence reads on the individual virus. Many reads were mapped on the whole region of the virus without gaps, enabling us to obtain the nearly complete genome (Figs. 5 and 6). Most viruses such as SPVF, SPVG, SPLV, SPFMV, and SPVC showed that the number of mapped reads increased from the 5′ region to the 3′ region of the virus genome (Figs. 5 and 6). In particular, SPLCV DNA A having a circular DNA genome exhibited different mapping patterns from other viruses. For example, a relatively small number of reads were mapped on the 5′ and 3′ regions and the region between AV1 and AC3 (Fig. 5d). The number of mapped reads was reduced from the AC3 region to the AC4 region.

We next examined the number of SNPs in each virus genome (Fig. 6g). Three SPFMV isolates showed a large number of SNPs ranging from 512 (Isolate YJ) to 1,036 (Isolate YJ-B) followed by three SPVC isolates ranging from 345 (Isolate IS) to 399 (Isolate GS). SPLV isolate YJ-B had the smallest number of SNPs among the 12 virus genomes. We calculated the frequency of SNPs according to each virus genome (Fig. 6h). Again, three SPFMV isolates showed a high frequency of SNPs ranging from 4.71% (Isolate YJ) to 9.67% (Isolate YJ-B). SPVE, SPVF, and SPVG showed low frequencies of SNPs.

We examined the positions of SNPs in each virus genome (Figs. 5 and 6). In general, the identified SNPs were randomly distributed on the virus genome. In particular, two SPVG isolates had many SNPs on the 3′ end region of the virus.

### Development of molecular methods to diagnose viruses infecting sweet potato

**Figure 7:**
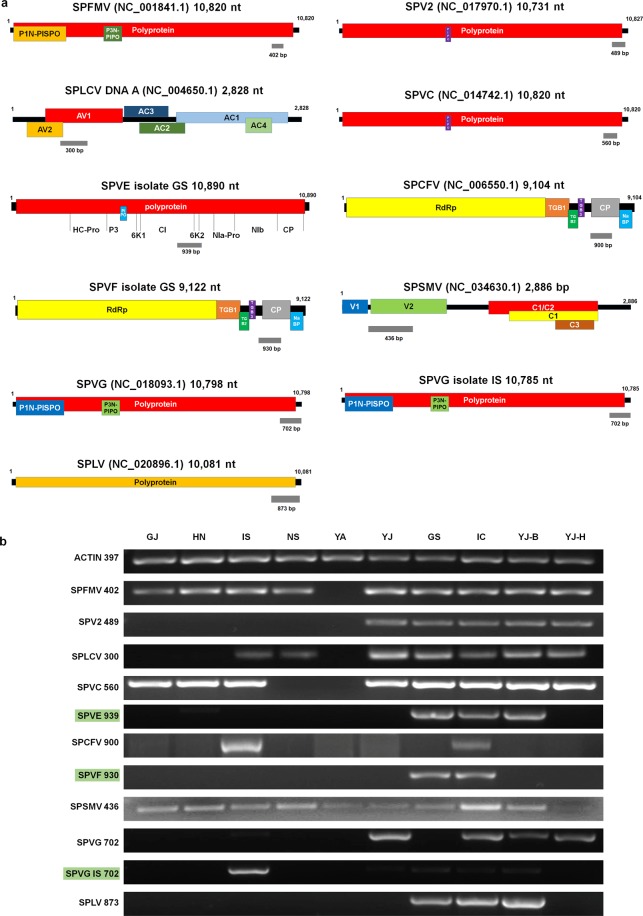
Confirmation of 11 identified viruses infecting sweet potato by RT-PCR. **(a)** Position of amplicon on individual virus genome was indicated by gray bar with respective size of amplicon. The detailed information of primer pairs can be found in Table S3. **(b)** Agarose gel electrophoresis results by RT-PCR with newly designed primer pairs. Full-length gels of RT-PCR results can be found in Fig. S1 in the Supplementary Information. *Actin* gene of sweet potato was used as positive control. We used the same total RNA for both NGS and RT-PCR. Green color indicates RT-PCR primer pairs for two novel viruses, SPVE and SPVF, as well as a variant of SPVG.

In this study, we identified a total of 10 different viruses infecting sweet potato. Based on the virus-associated reads in each library, SPCFV (16 reads) was identified from only one library (IS), while SPFMV (198,658 reads) was identified from eight libraries (Table 6). It is important to confirm RNA-Seq results and to develop molecular diagnostic methods for viruses infecting sweet potato. For that, we designed primer pairs for 10 viruses. RT-PCR primers for nine viruses were designed for RNA viruses with a single RNA genome, while PCR primers were designed for SPLCV with a single circular DNA genome based on the obtained virus sequences (Table S2 and Fig. 7a). A primer pair amplifying a partial *actin* gene was used as a positive control. We extracted total RNA and DNA from the same samples used for RNA-Seq. Amplified PCR products from each sample were visualized by gel electrophoresis (Figs. 7b and S1). In general, PCR results were correlated with those of RNA-Seq (Fig. 7b). For example, SPFMV was identified from nine libraries (all except YA) by RT-PCR, whereas SPFMV was identified from eight libraries (all except GS and YA) by RNA-Seq (Fig. 7b). In general, the PCR and RNA-Seq results were identical for SPV2, SPLCV, SPVF, SPSMV, and SPLV. In the case of SPVC, SPVE, and SPCFV, RT-PCR identified an additional virus infection compared to RNA-Seq. Based on genome sequence and phylogenetic analyses, SPVG isolate IS was distantly related with the other SPVG isolates. Therefore, we designed an additional primer pair for SPVG isolate IS. The primer pair for SPVG IS with a size of 702 bp could amplify SPVG isolate IS with high specificity.

**Table 6:** Information of virus-associated reads for viruses identified in sweet potato in Korea.

Index	Abbreviation	GJ	GS	HN	IC	IS	NS	YA	YJ	YJ-B	YJ-H
1	SPCFV					16					
2	SPFMV	60		191	26392	2020	1902		13046	104115	50932
3	SPLV		3359		1582					6365	
4	SPLCV		34027		4580	167	1518		4358	22140	17575
5	SPV2		3684		434				186	1737	5122
6	SPVC	1022	130595	1284		10400			2332	29625	27717
7	SPVG				841	4109			16331	317	3520
8	SPSMV	59	332	19	141	61	80	191	63	187	
9	SPVF		667		292						
10	SPVE		129306		49226						

To calculate the number of virus-associated reads, raw sequence reads were mapped on the identified virus genome using the BBMap program.

## Discussion

The word “virome” is derived from two words, “virus” and “genome,” which suggests it is similar to a viral genome^28^. Strictly speaking, a virome should be defined as all virus-associated nucleic acids existing in a specific organism, tissue, or environment^29^. A virome study should include viral genome information for not only a single virus but also multiple viruses. Moreover, a virome study reveals not only the presence of viruses but also the complexity of viral genomes, viral replication, viral mutation, and the change of viral genomes in a certain condition, as shown previously^24,26,27^.

Research associated with viruses infecting sweet potato has been intensively conducted in many countries^14,17,23,30^. However, there have been few comprehensive studies associated with sweet potato viromes. For instance, a previous study examined sweet potato viromes in three different sweet potato cultivars in China by NGS, revealing a total of 15 different viruses infecting sweet potato^21^. In addition, a large-scale sweet potato virome study is currently being conducted by the small RNA-Seq of 1,750 sweet potato samples collected from 12 countries in Africa, where the sweet potato is one of the main crops (http://bioinfo.bti.cornell.edu/virome/index).

In Korea, several studies have been carried out to understand viruses infecting sweet potato. For example, a nationwide survey was carried out to examine viruses infecting sweet potato from 2011 to 2014^14^. Based on multiplex RT-PCR assays, there were at least eight different viruses infecting sweet potato in Korea^16^. Furthermore, viral genomes of 18 isolates representing five potyviruses in Korea were determined^15^. As compared to previous studies associated with viruses infecting sweet potato in Korea, our sweet potato virome study provides valuable additional information associated with viruses infecting sweet potato in Korea. We revealed a total of 10 different sweet potato viromes representing eight different geographical regions and two major sweet potato cultivars in Korea. Our study demonstrated that each geographical region and cultivar displayed a unique sweet potato virome composed of different viruses, as shown in other plant viromes^27,31^. For instance, three viruses were identified from the GJ, HN, and NS regions; however, SPVC was dominantly present in GJ and HN, while SPFMV was the dominant virus in NS. The high proportion of SPLCV, which is transmitted by the whitefly, in the NS region compared to other regions suggests that insect vectors could be environmental factors determining the individual sweet potato virome. Samples for YJ-B and YJ-H were collected from the same region, Yeoju; however, the lists and proportions of infected viruses were very different, showing cultivar-specific sweet potato viromes.

Here, we identified a total of 10 different virus species infecting sweet potato. Our RNA-Seq-based approaches identified novel viruses and new virus variants more successfully than a PCR-based viral genome study, which could amplify highly conserved viral sequences^15^. In particular, the rare similarity of the 5′ regions in SPVE and SPVF with other homologous viruses highlights the superiority of NGS techniques followed by bioinformatics analyses. Although SPVF in the genus *Carlavirus* was closely related to SPCFV, the BLAST results and phylogenetic analyses revealed that SPVF is a new species. Two SPVF isolates, GS and IC, in the same group were different from other SPCFV isolates. Moreover, our phylogenetic analyses demonstrated that *Sweet potato yellow mottle virus* (SPYMV) isolate Yeongdeok (KR072674.1) should be a member of SPCFV. Moreover, two SPVE isolates, GS and YJ-B, were closely related to SPVC. However, the phylogenetic tree using polyprotein sequences of potyviruses infecting sweet potato demonstrated that SPVE was a new species in the genus *Potyvirus*.

Our sweet potato virome study indicated that SPFMV was the dominant virus followed by SPVC and SPVE in the examined regions in Korea. In addition, these three viruses are regarded as the main viruses infecting sweet potato in the world. We identified all previously reported viruses infecting sweet potato in Korea except *Sweet potato golden vein-associated virus* (SPGVaV) in the genus *Begomovirus*, which is rare in Korea^16,32^. Moreover, we did not identify SPCSV in the genus *Crinivirus*, which has been reported in many countries including China and Uganda^33,34^. Therefore, it is important to prevent the introduction of SPCSV to Korea.

Plant tissues and developmental stages are important factors for plant virome studies. Our previous studies demonstrated that viral RNA was enriched in grape fruits and lily flowers^24,35^. Of course, the optimal tissues for plant virome studies depend on the plant species. In our study, the proportion of virus-associated reads in the whole transcriptome was very low, indicating that leaf tissues might not be appropriate samples for the detection of viruses infecting sweet potato. As shown in a previous study^36^, we carefully suggest using samples from the fibrous and tuberous roots of sweet potato for virus detection. Based on our experience studying viruses infecting plants with a large genome size such as the hexaploid sweet potato (4.4 Gb)^36^, selection of the proper tissue enriched with viruses is necessary for a successful virome study.

In this study, we used messenger RNA from total RNA for the library preparation using oligo-d(T). Eight RNA viruses in this study possessed polyadenylate (poly(A)) tails (all except SPLCV and SPSMV). As shown in other previous studies^21,35^, virus genomes with poly(A) tails can be easily assembled. It is also not surprising that genomes of DNA viruses such as SPLCV can also be assembled from transcriptome data, as shown in our previous studies^26^. In many cases, the number of mapped sequence reads on the viral genomes with poly(A) tails was increased from the 5′ region to the 3′ region. This result is somehow correlated with the result of poly(A) tails preferentially attaching to the transcripts close to the poly(A) tails.

We examined the mutation rates for the 12 assembled virus isolates. Of them, SPFMV showed a high mutation rate of up to 9.67%. Furthermore, a recent study has found possible recombination within the Nla-Pro, CP, and P1 genes using available SPFMV genomes^30^. In addition, SPLCV also exhibited a high frequency of mutations of up to 4.52%. Similarly, several Korean SPLCV recombinants have been identified^13^. Thus, our result suggests that mutation and recombination contribute to the genetic diversity of SPFMV and SPLCV isolates. Although several potyviruses were coinfected, the mutation rate was the highest in SPFMV followed by SPVC, SPVG, and SPLV. SPLV showed the lowest mutation rate among the examined potyviruses. This result suggests SPFMV might play an important role in viral disease symptoms in coinfected sweet potato plants.

Here, we examined sweet potato viromes for two popular sweet potato cultivars in Korea, Beni Haruka and Hogammi. Beni Haruka originates from Japan, while Hogammi was recently developed by the Rural Development Administration (RDA) in Korea. As they are popular for roasting, many growers cultivate both cultivars. In the case of Beni Haruka, at least seven different viruses were identified, whereas five different viruses were identified from Hogammi. Interestingly, both cultivars were cultivated in the same field in Yeoju, Korea; however, Beni Haruka was more severely infected by viruses than Hogammi. The difference in the number of infected viruses between the two cultivars might be correlated with the cultivation period. That is, Beni Haruka has been cultivated for a long time, while Hogammi was introduced to Korean growers only three years ago by the RDA.

It is now possible to *de novo* assemble many viral genomes from transcriptome data^37^. Similarly, we obtained 12 complete virus genomes and 18 nearly complete genomes for eight viruses. A total of 30 assembled viral genomes were further used for phylogenetic analyses. Our phylogenetic analyses showed that many isolates of SPVC, SPLV, SPV2, and SPLCV in this study were grouped together with other known isolates from Korea. In general, it is likely that virus genomes are highly correlated with geographical regions. However, two isolates of SPFMV and a single isolate of SPVG were genetically distant from other known isolates. For example, two SPFMV isolates, YJ-H and YJ-B, were revealed as common ancestors of other known SPFMV isolates. Furthermore, SPVG isolate IS in this study and SPVG isolate WT325 from Taiwan were genetically distant from other SPVG isolates, suggesting they are new variants of SPVG.

As shown in other previous studies, the coinfection of multiple viruses in sweet potato resulted in the dramatic reduction of sweet potato production by up to 50%^38^. Unfortunately, most sweet potato cultivars in Korea were severely coinfected by many viruses^14^. There are several possible reasons explaining how viruses infect sweet potato plants. The first is vegetative propagation. Most growers purchase sweet potato sprouts from seedling markets, and they are often already infected by diverse viruses. Surprisingly, a recent study showed that most sweet potato germplasm (83.8%) in the Bioenergy Research Center of the RDA in Korea, which is used to develop new cultivars or provide sweet potato cuttings, was already infected by multiple viruses^14^. Furthermore, a recent study demonstrated that SPLCV can be transmitted by seeds, suggesting the newly developed sweet potato cultivars might also be highly infected by viruses^8^. Thus, it seems that the sweet potato virus control in Korea should be conducted from the early stages of breeding.

Based on the above evidence, it is necessary to develop virus-free sweet potato cultivars in order to prevent the damages caused by viral diseases, as suggested previously^38^. For that, knowledge of the viruses infecting sweet potato and diagnostic methods are needed. In this study, we provide a complete list of viruses infecting sweet potato in Korea and diagnostic methods, which could be valuable information for the development of a virus-free sweet potato in Korea in the near future. Moreover, we suggest that the development of a virus-free sweet potato in Korea should be carried out by not only national institutes such as the RDA but also universities and companies. We have to provide various reasonable choices to growers to promote competition among different developers of virus-free sweet potatoes. Dependence on a single national institute does not guarantee the production of a high-quality virus-free sweet potato, as we have seen.

## Methods

### Collection of sweet potato samples

We collected sweet potato leaf samples from eight different geographical regions in Korea in May 2016 and May 2017 (Table 1 and Fig. 1a). The eight regions are the main sweet potato producing areas in Korea. Most collected samples showed viral disease symptoms; however, we also collected leaf samples without any visible disease symptoms. In order to examine viruses infecting sweet potato in different geographical regions, leaf samples were pooled according to geographical regions and used for total RNA extraction. In addition, leaf samples from two major sweet potato cultivars referred to as “Beni Haruka” and “Hogammi” were also collected to compare sweet potato viromes in different sweet potato cultivars. As a result, a total of 357 samples were used for 10 different libraries.

### Total RNA extraction and library preparation for RNA-Seq

Leaf samples were pooled and then frozen in the presence of liquid nitrogen. The frozen leaf samples were ground with a pestle and mortar. We used the RNeasy Plant Mini Kit (Qiagen, Hilden, Germany) to extract total RNA for RNA-Seq based on the manufacturer’s instructions. The quality and quantity of extracted total RNA were measured by gel electrophoresis followed by using an Agilent 2100 Bioanalyzer (Agilent, Santa Clara, CA, U.S.A.). Using the extracted total RNA, we generated 10 different RNA-Seq libraries using the NEBNext Ultra RNA Library Prep Kit for Illumina in accordance with the manufacturer’s instructions (NEB, Ipswich, Massachusetts, U.S.A.). Briefly, poly(A)-tailed mRNA was extracted by using poly-T oligo-attached magnetic beads. We synthesized the first strand of cDNA from the extracted mRNA followed by a second strand of cDNA. The 3′ ends of the DNA fragments were adenylated. After adapter ligation, we performed PCR amplification to selectively enrich DNA fragments with adapters and amplify the large amount of DNA in the library. We again measured the quality and quantity of each library using the 2100 Bioanalyzer. The six prepared libraries from 2016 were paired-end sequenced by Theragen (Suwon, South Korea) using the HiSeq2300 platform, and the four libraries from 2017 were paired-end sequenced by Macrogen Co. (Seoul, South Korea) using the HiSeq2000 platform.

### *De novo* transcriptome assembly and virus identification

In the bioinformatics analyses including the *de novo* transcriptome assembly and BLAST search, a workstation with two 20-core CPUs and 256 GB of RAM installed with Ubuntu 16.04.4 LTS was used. Based on the results of our previous studies^31^, we only used the Trinity program (version 2.0.2, released January 22, 2015) with default parameters for *de novo* transcriptome assembly^39^. Raw sequence reads from each library were *de novo* assembled using Trinity. We generated our own plant viral genome database by selecting only viruses infecting plant species from the viral reference database of the NCBI (https://www.ncbi.nlm.nih.gov/genome/viruses/). To identify virus-associated contigs, the assembled contigs (transcriptome) from each library were blasted against the plant viral genome database using MEGABLAST^40^ with a cutoff E-value of 1e–6. The obtained virus-associated contigs were again blasted against NCBI’s NT database to remove sweet potato host sequences and other contaminated sequences. Ultimately, only virus-associated contigs were used for the study of sweet potato viromes. To calculate the number of virus-associated reads for identified viruses, the BBMap program was used (https://sourceforge.net/projects/bbmap/).

### Virus genome assembly and annotation

Based on the BLAST results, the virus-associated contigs in each library were aligned on the identified virus reference genomes using the ClustalW program implemented in the MEGA7 program^41^. Several nearly complete viral genome sequences were obtained without any further alignment. Poly(A) tail sequences at the 3′ terminal were deleted. We again aligned raw sequence reads on the identified virus genome sequences to fill the missing gaps of the virus genome using a Burrows–Wheeler Aligner (BWA) program with default parameters^42^. To predict the ORFs in each virus genome, we used the ORF Finder program (https://www.ncbi.nlm.nih.gov/orffinder/). In addition, we manually checked the identified ORFs and the 5′ and 3′ untranslated regions (UTRs) by comparing the corresponding reference virus genome. Twelve complete viral genome sequences covering whole ORFs were deposited in NCBI’s GenBank database with respective accession numbers (Table 5).

### Phylogenetic analyses of identified viruses

For phylogenetic tree analyses, we used the virus genome sequences assembled in this study (Table S2) as well as the available viral genome sequences from GenBank for eight viruses. We used RdRp amino sequences, CP amino sequences, and a complete genome sequence for SPVF and its homologous sequences, whereas we used a polyprotein amino acid sequence and a complete genome sequence for SPVE and its homologous sequences. In the case of six viruses (i.e., SPFMV, SPVC, SPVG, SPLV, SPV2, and SPLCV), complete genome sequences obtained in this study as well as available complete viral genome sequences from GenBank were used. Six SPFMV genomes, seven SPVC genomes, two SPVG genomes, three SPLV genomes, four SPV2 genomes, and four SPLCV genomes were used for the construction of phylogenetic trees in this study. For the individual virus, viral sequences were aligned using the ClustalW program. The aligned nucleotide sequences or amino acid sequences were used for phylogenetic tree construction using the MEGA7 program with the maximum likelihood method and 1,000 bootstrap replicates^41^.

### Identification of SNPs for 12 assembled virus genomes

For virus SNP identification, it is important to use the assembled virus genome sequences in each library as reference virus genome sequences to increase SNP specificity. We analyzed single SNPs for the 12 assembled virus genomes as described previously^27^. The raw sequence reads in individual libraries were aligned on the assembled viral genome using the BWA program with default parameters, resulting in the Sequence Alignment Map (SAM) files. The SAM files were converted into Binary Alignment Map (BAM) files using SAMtools^43^. After that, the sorted BAM files were used to generate the Variant Call Format (VCF) file format using the mpileup function of SAMtools for SNP calling. Finally, we called SNPs using BCFtools implemented in SAMtools. The positions of identified SNPs and mapped reads on each viral genome were visualized by the Tablet program^44^.

### RT-PCR assay

In order to confirm the results of RNA-Seq, we carried out RT-PCR. For that, we designed 12 RT-PCR primer pairs. An *actin* gene of sweet potato was used as a positive control. In the case of SPVG, two different primer pairs, SPVG and SPVG_IS, were designed. The SPVG_IS-specific primer pair can amplify the variant of SPVG identified from the IS region. Detailed information of designed primer pairs can be found in Table S3. The regions of each virus amplified by RT-PCR can be found in Fig. 7a. We used the same total RNA from the pooled samples as a template RNA for the RT-PCR assay. RT-PCR was performed using the DiaStar OneStep RT-PCR Kit (SolGent, Daejeon, Korea). As described previously^31^, the RT-PCR conditions were 50 °C for 30 min, 95 °C for 15 min, followed by 30 cycles at 95 °C for 20 sec, 50 °C to 56 °C for 40 sec (the annealing temperature can be varied depending on the Tm values of primers), and 72 °C for 1 min, with a final extension at 72 °C for 5 min. We checked the amplified RT-PCR products by gel electrophoresis followed by EtBr staining. Furthermore, we cloned the amplified RT-PCR product in the pGEM-T-Easy Vector (Promega, Wisconsin, US) followed by Sanger sequencing to confirm the sequences of amplified PCR products.

To confirm complete genome sequences of SPVE and SPVF, we carried out RT-PCR using newly designed primers (Table S4 and Fig. S2). The amplified RT-PCR products were visualized by gel electrophoresis (Fig. S2). We confirmed amplicon sequences by cloning into the pGEM-T-Easy Vector and Sanger sequencing.

## Supplementary information

Supporting information.

Table S1.

Table S2.

Table S3.

Table S4.

## Data Availability

The raw dataset in this study will be available, upon publication, in the Sequence Read Archive (SRA) repository with accession numbers SRR8489804, SRR8489848, SRR8492257, SRR8492258, SRR8492261, SRR8492260, SRR8492261, SRR8492262, SRR8492263, and SRR8492264. The 12 viral genome sequences obtained from this study were also deposited in GenBank, NCBI, with respective accession numbers.

## References

[1] O’brien PJ (1972). The Sweet Potato: Its Origin and Dispersal 1. Am. anthropologist.

[2] Drapal M, Rossel G, Heider B, Fraser PD (2019). Metabolic diversity in sweet potato (Ipomoea batatas, Lam.) leaves and storage roots. Horticulture Res..

[3] Bovell‐Benjamin AC (2007). Sweet potato: a review of its past, present, and future role in human nutrition. J. Adv. food.

[4] Megersa HJJH (2017). Propagation Methods of Selected Horticultural Crops by Specialized Organs: Review. J. Horticulture.

[5] No S-H (2010). Joseon Tongsinsa and the introduction of sweet patato. J. OF. NORTH-EAST ASIAN CULTURES.

[6] Clark CA (2012). Sweetpotato viruses: 15 years of progress on understanding and managing complex diseases. Plant. Dis..

[7] Colinet D, Kummert J, Lepoivre P, Semal J (1994). Identification of distinct potyviruses in mixedly-infected sweetpotato by the polymerase chain reaction with degenerate primers. Phytopathology.

[8] Kim J (2015). Seed transmission of Sweet potato leaf curl virus in sweet potato (Ipomoea batatas). Plant. Pathol..

[9] Wosula E, Davis J, Clark C (2014). Stylet penetration behaviors of Myzus persicae (Hemiptera: Aphididae) on four Ipomoea spp. infected or noninfected with sweet potato potyviruses. J. Economic Entomology.

[10] Mulabisana, M. *et al*. Yield evaluation of multiple and co-infections of begomoviruses and potyviruses on sweet potato varieties under field conditions and confirmation of multiple infection by NGS. *Crop Protection* (2019).

[11] Karyeija R, Kreuze J, Gibson R, Valkonen J (2000). Synergistic interactions of a potyvirus and a phloem-limited crinivirus in sweet potato plants. Virology.

[12] Mukasa SB, Rubaihayo PR, Valkonen J (2006). Interactions between a crinivirus, an ipomovirus and a potyvirus in coinfected sweetpotato plants. Plant. Pathol..

[13] Kim J (2018). Phylogeographic analysis of the full genome of Sweepovirus to trace virus dispersal and introduction to Korea. PLoS one.

[14] Kim J (2017). Virus Incidence of Sweet Potato in Korea from 2011 to 2014. Plant. Pathol. J..

[15] Kwak H-R (2015). Molecular characterization of five potyviruses infecting Korean sweet potatoes based on analyses of complete genome sequences. Plant. Pathol. J..

[16] Kwak H-R (2014). The current incidence of viral disease in Korean sweet potatoes and development of multiplex RT-PCR assays for simultaneous detection of eight sweet potato viruses. Plant. Pathol. J..

[17] Nhlapo T, Rees D, Odeny D, Mulabisana J, Rey M (2018). Viral metagenomics reveals sweet potato virus diversity in the Eastern and Western Cape provinces of South Africa. South. Afr. J. Botany.

[18] Maree HJ, Fox A, Al Rwahnih M, Boonham N, Candresse T (2018). Application of HTS for routine plant virus diagnostics: state of the art and challenges. J. Front. Plant. Sci..

[19] Jones S, Baizan-Edge A, MacFarlane S, Torrance L (2017). Viral diagnostics in plants using next generation sequencing: computational analysis in practice. Front. Plant. Sci..

[20] Pooggin, M. M. Small RNA-Omics for Plant Virus Identification, Virome Reconstruction, and Antiviral Defense Characterization. *Frontiers in Microbiology* **9** (2018). 10.3389/fmicb.2018.02779 PMC6256188 30524398

[21] Gu Y-H, Tao X, Lai X-J, Wang H-Y, Zhang Y-Z (2014). Exploring the polyadenylated RNA virome of sweet potato through high-throughput sequencing. PLoS One.

[22] Mbanzibwa D, Tugume A, Chiunga E, Mark D, Tairo F (2014). Small RNA deep sequencing‐based detection and further evidence of DNA viruses infecting sweetpotato plants in Tanzania. Ann. Appl. Biol..

[23] Ma S (2019). Identification of viruses infecting sweet potato in southern China by small RNA deep sequencing and PCR detection. J. Gen. Plant. Pathol..

[24] Jo Y (2015). In silico approach to reveal viral populations in grapevine cultivar Tannat using transcriptome data. Sci. Rep..

[25] Jo Y (2016). Integrated analyses using RNA-Seq data reveal viral genomes, single nucleotide variations, the phylogenetic relationship, and recombination for Apple stem grooving virus. BMC genomics.

[26] Jo Y (2017). The pepper virome: natural co-infection of diverse viruses and their quasispecies. BMC genomics.

[27] Jo Y (2018). Peach RNA viromes in six different peach cultivars. Sci. Rep..

[28] McDaniel L (2008). Metagenomic analysis of lysogeny in Tampa Bay: implications for prophage gene expression. PLoS One.

[29] Breitbart M (2002). Genomic analysis of uncultured marine viral communities. Proc. Natl Acad. Sci..

[30] Wainaina JM, Ateka E, Makori T, Kehoe MA, Boykin LM (2018). Phylogenomic relationship and evolutionary insights of sweet potato viruses from the western highlands of Kenya. PeerJ.

[31] Jo, Y. *et al*. Barley RNA viromes in six different geographical regions in Korea. *Scientific Reports* **8** (2018). 10.1038/s41598-018-31671-4 PMC6125401 30185900

[32] Kil E-J (2014). First Report of Sweet potato golden vein associated virus Infecting Sweet Potato in Korea. Plant. Dis..

[33] Qin Y (2014). Complete genomic sequence and comparative analysis of the genome segments of Sweet potato chlorotic stunt virus in China. PLoS One.

[34] Tugume AK (2013). Genetic variability and evolutionary implications of RNA silencing suppressor genes in RNA1 of Sweet potato chlorotic stunt virus isolates infecting sweetpotato and related wild species. PLoS One.

[35] Jo Y, Cho WK (2018). RNA viromes of the oriental hybrid lily cultivar “Sorbonne”. BMC Genomics.

[36] Yang J (2017). Haplotype-resolved sweet potato genome traces back its hexaploidization history. Nat. plants.

[37] Blawid R, Silva J, Nagata T (2017). Discovering and sequencing new plant viral genomes by next‐generation sequencing: description of a practical pipeline. Ann. Appl. Biol..

[38] Loebenstein, G. In *Advances in virus research* Vol. 91, 33–45 (Elsevier, 2015). 10.1016/bs.aivir.2014.10.005 25591876

[39] Grabherr MG (2011). Full-length transcriptome assembly from RNA-Seq data without a reference genome. Nat. Biotechnol..

[40] Morgulis A (2008). Database indexing for production MegaBLAST searches. Bioinforma..

[41] Kumar S, Stecher G, Tamura K (2016). MEGA7: Molecular Evolutionary Genetics Analysis version 7.0 for bigger datasets. Mol. Biol. Evol..

[42] Li H, Durbin R (2009). Fast and accurate short read alignment with Burrows–Wheeler transform. Bioinforma..

[43] Li H (2009). The sequence alignment/map format and SAMtools. Bioinforma..

[44] Milne I (2012). Using Tablet for visual exploration of second-generation sequencing data. Brief. Bioinform..

