# Supplementary material for: Sweet potato viromes in eight different geographical regions in Korea and two different cultivars
Source: Sci Rep. 2020 Feb 13;10:2588. doi: 10.1038/s41598-020-59518-x (PMC7018812; doi:10.1038/s41598-020-59518-x)

**Supporting information**

**Sweet potato viromes in eight different geographical regions in Korea and two different cultivars**

Yeonhwa Jo^1,#^, Sang-Min Kim^2,#^, Hoseong Choi^3,#^, Jung Wook Yang^4^, Bong Choon Lee^2,*^, and Won Kyong Cho^1,*^

^1^Research Institute of Agriculture and Life Sciences, College of Agriculture and Life Sciences, Seoul National University, Seoul 08826, Republic of Korea

^2^Crop Foundation Division, National Institute of Crop Science, Rural Development Administration, Wanju 55365, Republic of Korea

^3^Department of Agricultural Biotechnology, College of Agriculture and Life Sciences, Seoul National University, Seoul 08826, Republic of Korea

^4^Bioenergy Crop Research Institute, National Institute of Crop Science, Rural Development Administration, Muan 58545, Republic of Korea

**Supplementary data**

**Supplementary Tables**

**Table S1**. Sequence identity of individual open reading frames (ORFs) for two novel viruses, SPVE and SPVF, with known viral sequences.

In order to determine novel viral species, we examined sequence identities of two novel viruses.

For that, each ORF protein was subjected to BLASTP search against NCBI NR database.

Individual viral ORF and its highly homologous viral sequence were subjected to Clustal Omega (https://www.ebi.ac.uk/Tools/msa/clustalo/) for the calculation of sequence identity.

Species demarcations criteria for *potyvirus* genus complete ORF are <76% nucleotide identity and <82% amino acid identity.

Species demarcation for carlavirus is 72% nt identity (or 80% aa identity) between their respective CP or polymerase genes.

**Table S2.** Complete or nearly complete viral genome sequences were used for phylogenetic analyses in this study.

Name of each virus genome, isolate name, and size of genome were indicated. “not_full_seq” indicates incomplete virus genome sequence. Missing sequences were indicated with “N”.

**Table S3.** Detailed information on RT-PCR primer pairs to detect 11 viruses infecting sweet potato.

**Table S4.** Detailed information on RT-PCR primer pairs to confirm complete genome sequences of SPVE and SPVF.

**Supplementary Figure**

**Fig. S1.** Full-length gels display RT-PCR results with virus-specific primer pairs. *Actin* gene of sweet potato was used as positive control.


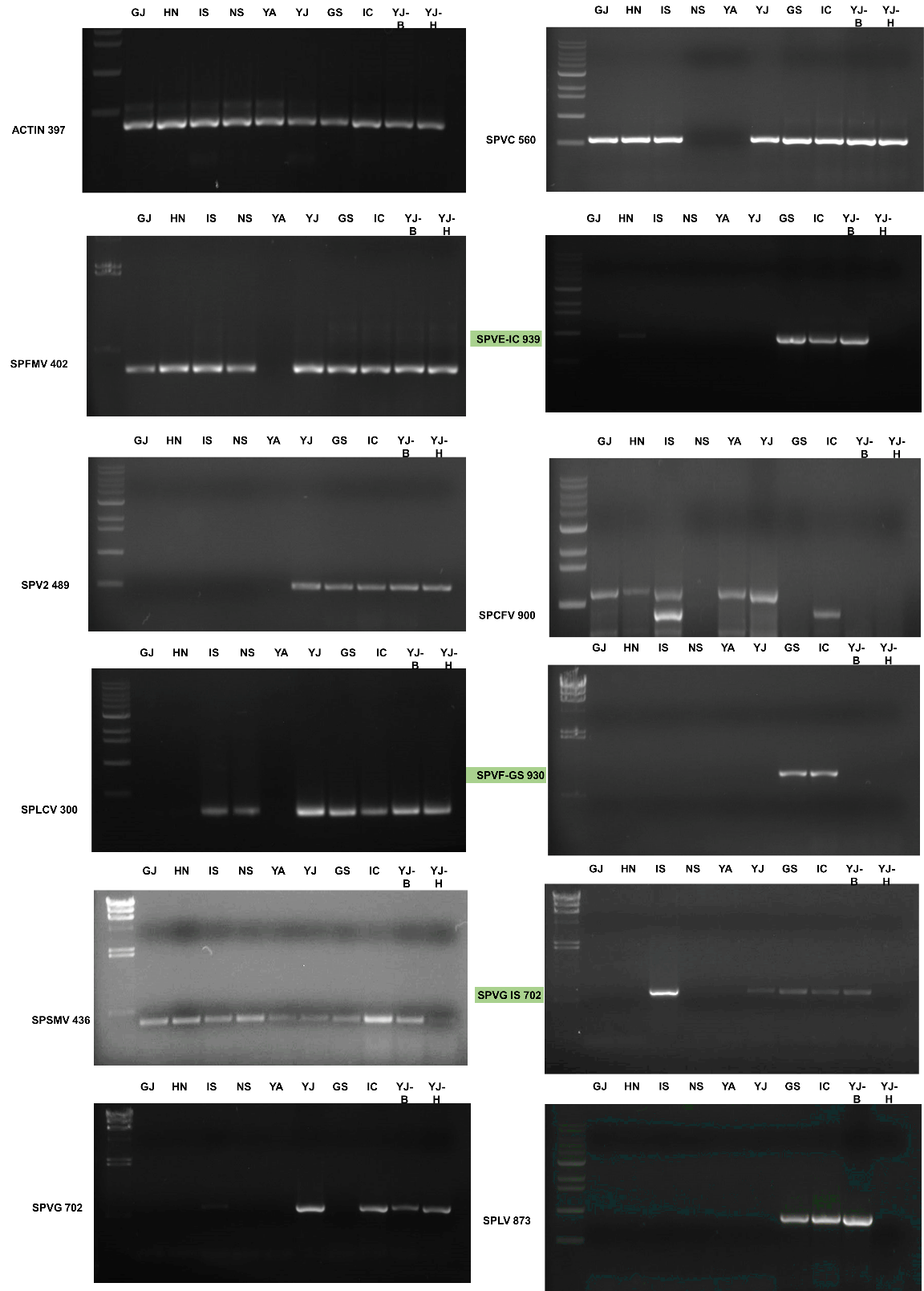


**Fig. S2.** Confirmation of genome sequences of SPVE and SPVF by RT-PCR.

Genome organizations of SPVE (a) and SPVF (b) with RT-PCR primers. Red arrows indicate primer positions with primer names. Full-length gels display amplicons by RT-PCR for SPVE (c) and SPVF (d). Detailed information for RT-PCR primers can be found in Table S4.


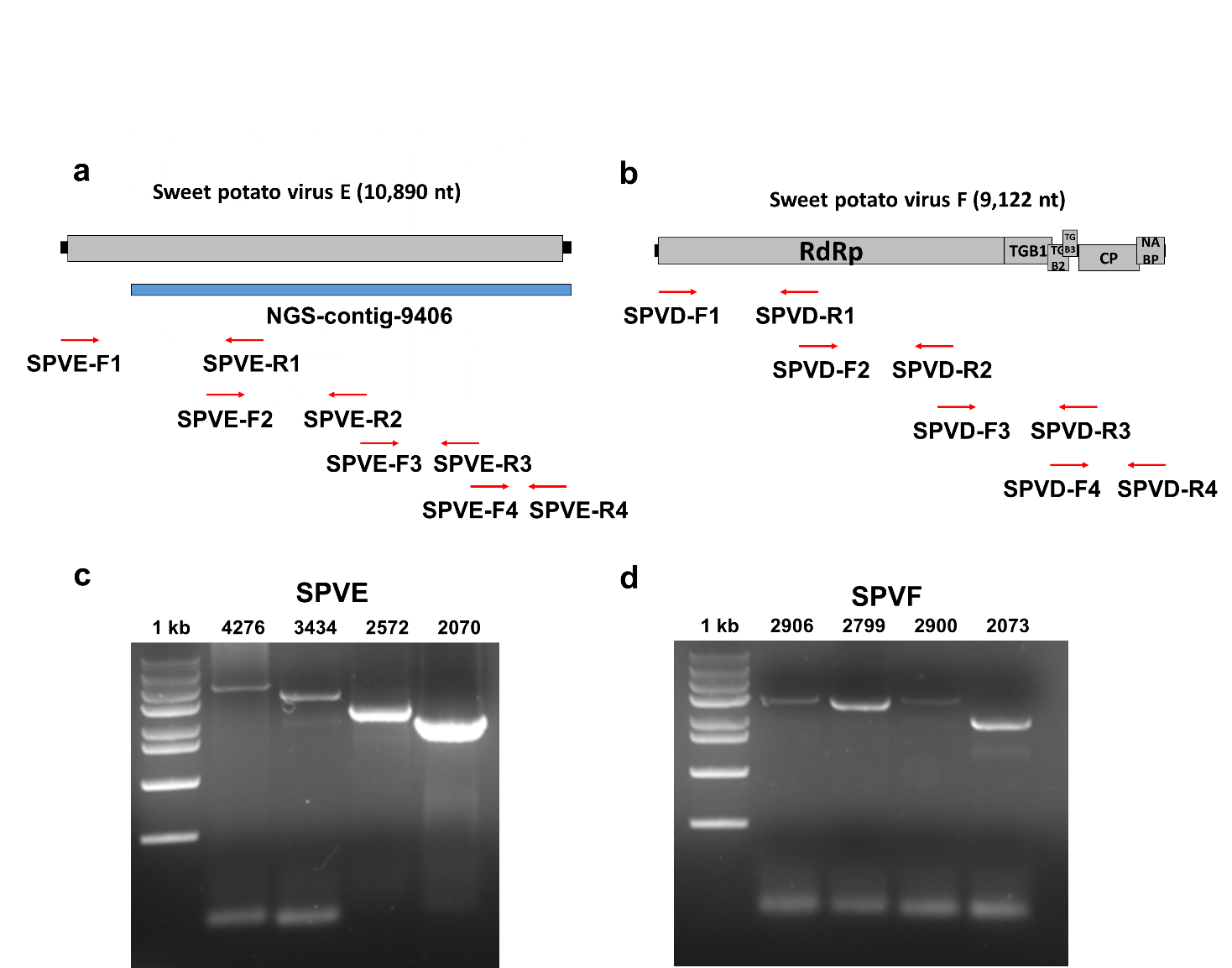

Supplement: Supplementary file 1 — Supporting information. [file 41598_2020_59518_MOESM1_ESM.docx]
